# False Positives in Esophageal pH Monitoring: The Pitfalls of Single-Value Interpretation

**DOI:** 10.3390/diagnostics16203306

**Published:** 2026-10-12

**Authors:** Kyle Nguyen-Ngo, Kumael Jafri, Vinit H. Majmudar, Rajsri Bipat, Wanda Sykes, John E. Pandolfino, Micheal Tadros

**Affiliations:** 1 Albany Medical College, Albany, NY 12208, USA; nguyenky@amc.edu (K.N.-N.); kumael.jafri@baystateheatlh.org (K.J.); vinmajcreates@gmail.com (V.H.M.); 2 Albany Medical Center, Department of Gastroenterology, Albany, NY 12208, USA; bipatr@amc.edu (R.B.); sykesw@amc.edu (W.S.); 3 Kenneth C. Griffin Esophageal Center of Northwestern Medicine, Department of Medicine, Division of Gastroenterology and Hepatology, Feinberg School of Medicine, Northwestern University, Chicago, IL 60611, USA; j-pandolfnio@northwestern.edu

**Keywords:** pH testing, gastroesophageal reflux disease, esophagus, acid exposure time, false-positive

## Abstract

**Background/Objectives**: Esophageal pH monitoring is a key diagnostic modality in the evaluation of gastroesophageal reflux disease (GERD) and employs either a traditional transnasal catheter or wireless probe. In clinical practice, findings from these studies are often represented by a single metric, such as acid exposure time (AET), designed to quantitatively summarize reflux burden. However, these composite scores are susceptible to error derived from sensor position, patient diet and posture, transient pH fluctuations, anatomical variations, and esophageal motility. This study sought to identify false-positive mechanisms in esophageal pH testing. **Methods**: We retrospectively identified institutional logs of false-positive pH findings from a single medical center from January 2019 to January 2025, and in conjunction with expert opinion, we classified false-positive patterns based on the original clinical interpretation. **Results**: The most common mechanism was technical error originating from factors that directly impact sensor integrity, such as a dislodged capsule or withdrawal of the transnasal catheter. Additional confounders comprised patient-specific variables, including esophageal stasis or behavioral disorders like belching or rumination syndrome. **Conclusions**: Ultimately, false-positive GERD findings are uncommon but can occur when AET is derived from misleading data. We also sought to develop a diagnostic framework that incorporates clinical context, adjunctive testing, and sensor verification protocols in order to mitigate confounders inherent to ambulatory pH monitoring. This study represents one of the first evaluations of false-positive patterns in esophageal pH testing, offering insight that may ultimately enhance precision and clinical decision-making in the diagnosis of GERD.

## 1. Introduction

Ambulatory pH monitoring is a cornerstone modality in diagnosing gastroesophageal reflux disease (GERD), which affects 20% of the United States population and carries long-term sequelae, including Barrett esophagus and esophageal adenocarcinoma [1,2,3]. Testing traditionally utilizes a transnasal catheter that is positioned at the proximal border of the lower esophageal sphincter (LES). Probe insertion is often aided by manometry, which allows clinicians to not only map key esophageal landmarks but also account for individual anatomic variability [4,5]. Various metrics, such as the quantity and duration of reflux events, are then continuously measured for 24 h while the patient maintains their day-to-day routine [4]. Wireless pH monitoring (e.g., Bravo study) offers an alternative that enhances both patient comfort and recording duration through the utilization of a capsule that is endoscopically attached to the lower esophageal mucosa [6,7].

While acid testing has historically been considered the gold standard for reflux studies, there exist several pitfalls that limit its clinical efficacy and interpretation [3,8]. A threshold of pH < 4 is traditionally employed but precludes weakly acidic or non-acidic reflux from being identified as pathologic events, despite their potential contribution to characteristic GERD symptoms [4,9,10]. Additionally, acid testing cannot directly gauge the presence or composition of refluxate and thus cannot differentiate between true reflux events and transient pH fluctuations unrelated to retrograde bolus movement [9]. These limitations pose significant issues in patients on proton pump inhibitors (PPIs), in which acid exposure may be suppressed, but refluxate volume can persist [11]. To that end, impedance testing, which is commonly performed as multichannel intraluminal impedance combined with pH monitoring (MII-pH), was designed to rectify the clinical pitfalls of traditional pH monitoring. Impedance testing employs electrodes to record the presence of gas and refluxate within the esophagus through shifts in luminal electrical resistance, allowing clinicians to detect both the proximal extent and composition of refluxate, as well as the full spectrum of acid exposure [12,13,14].

Regardless of the modality employed, study findings are often distilled into a single metric, such as acid exposure time (AET), in clinical practice [4,15]. AET is a cumulative metric that represents the percentage of the total recording length during which the distal esophageal pH is below 4. According to the Lyon Consensus, there exist distinct threshold values depending on PPI indication. An AET of greater than 6% strongly suggests pathologic GERD for patients off PPIs, which are typically held for 7–10 days prior to study onset, while an AET of less than 4% is considered physiologic and renders clinically significant reflux unlikely. A borderline AET between 4% and 6% requires adjunctive testing for conclusive interpretation. Conversely, among patients on PPIs, an AET of greater than 4% is indicative of poor acid control in refractory GERD. Since its inception in the 1970s, AET has emerged as a key determinant in not only establishing a diagnosis of GERD but also evaluating patients for anti-reflux intervention [15].

Despite its diverse utility, reliance on AET in isolation can oversimplify and potentially misrepresent the broader reflux profile and its clinical context, as both traditional pH testing and MII-pH possess structural limitations that contribute to diagnostic error [14]. One such pitfall is the inherent volatility of acid exposure, which is influenced by diet, body position, stress, and circadian variation. As such, patients are instructed to maintain a diary documenting symptom occurrence, meals, and activities, thereby allowing for contextual correlation of acid exposure patterns [16,17,18]. Data integrity is contingent upon precise sensor positioning, and anatomic variability (e.g., obesity, hiatal hernias) can lead to improper localization of the LES and subsequent probe misplacement [19,20]. In a similar manner, artifact may also stem from instrument error, as sensor migration or dislodgement can produce spurious fluctuations in recorded acid exposure independent of true reflux events [21].

Composite metrics offer structured summaries of reflux burden, but their diagnostic value relies upon appropriate clinical context. As pH monitoring is susceptible to technical and physiologic error, these scores should be corroborated by not only confirmatory studies like upper endoscopy or high-resolution impedance manometry (HRIM) but also relevant medical history and comorbid profile. This retrospective study of cataloged false positive mechanisms in esophageal pH monitoring highlights the pitfalls of construing composite scores as self-contained diagnostic endpoints and emphasizes the value of integrating these measures into the broader clinical picture.

## 2. Materials and Methods

From January 2019 to January 2025, false-positive cases from ambulatory pH studies were systematically cataloged from a single medical center for patients who underwent catheter-based, capsule-based, or esophageal impedance pH testing. Cases were retrospectively assessed for mechanisms of diagnostic error following identification of clinical discrepancies that had ultimately influenced study outcomes.

Subjects were adult patients (≥18 years old) who were referred for esophageal testing for any of the following indications: dysphagia, reflux, chest pain, or regurgitation. Patients under 18 years old, pregnant patients at the time of testing, studies performed for non-GERD indications, and incomplete or technically inadequate studies (e.g., patient intolerance, failed probe placement) that precluded interpretation were excluded from review.

This evaluation was performed in conjunction with expert consultation and pooled cases accumulated through clinical practice. False positive results were defined as an abnormal composite metric in the absence of corroborating evidence of GERD, and when an alternative technical, physiologic, or behavioral explanation for the positive finding was identified. The false positive mechanism was categorized for further analysis.

## 3. Results

**Figure 1:**
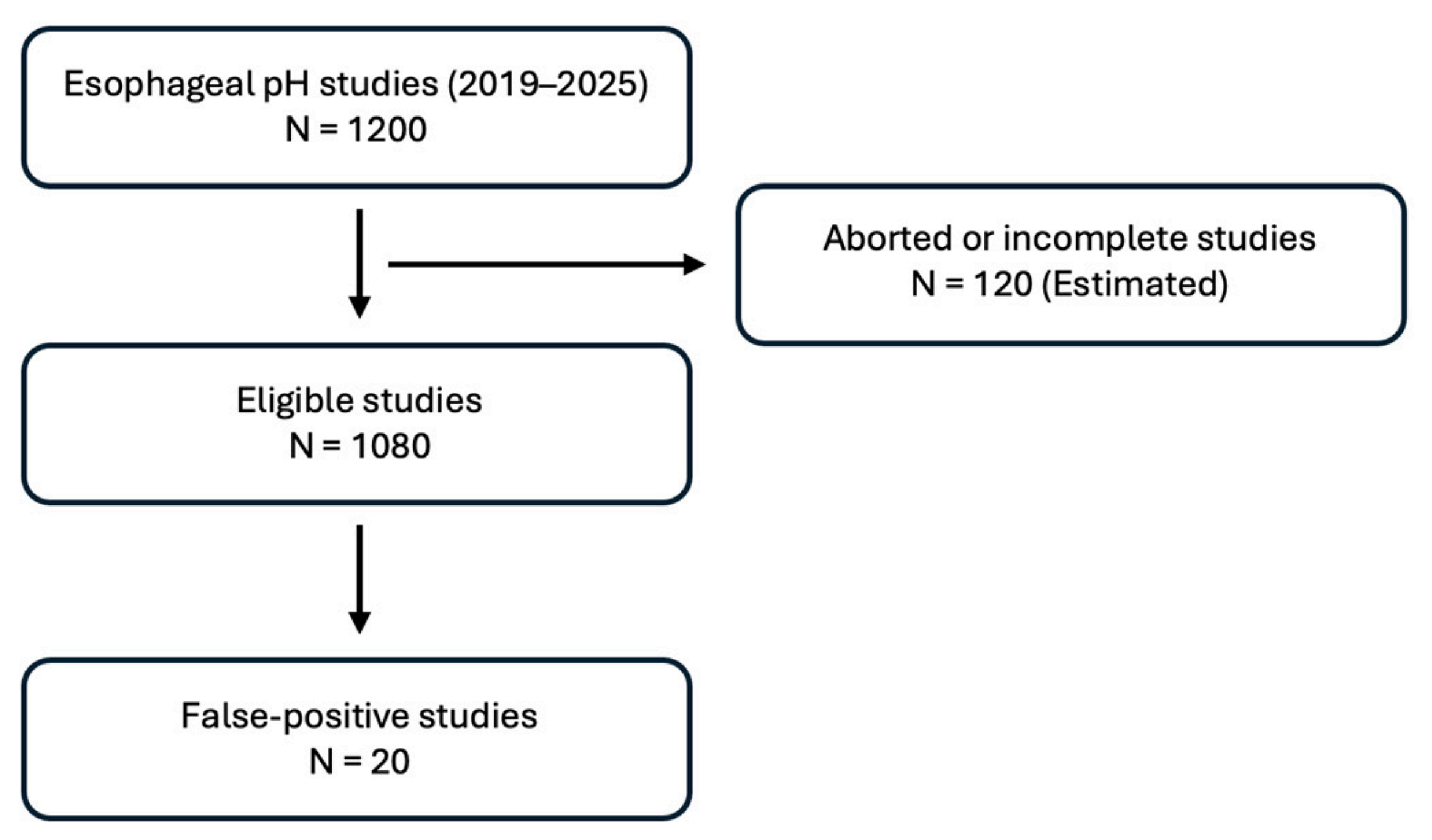
Flow diagram illustrating case identification for false-positive categorization.

A total of 1200 esophageal pH studies were performed during the specified timeframe. While cancelled cases are not reliably logged, an estimate of 120 studies were excluded per our selection criteria, resulting in 1080 finalized studies. We ultimately identified 20 cases in which an initial positive finding was later corrected through either repeat testing or re-assessment of the data (Figure 1). While this study quantified that 1.9% of all eligible cases during this timeframe yielded an initial false-positive reading, this figure is likely subject to reporting bias and is thus an underrepresentation of the true false-positive incidence. Importantly, the aim of this study was to simply characterize false-positive mechanisms that may occur in esophageal pH testing, rather than propose a false-positive rate, as patient compliance with PPI adherence, dosing, and washout timing was not consistently included in clinical notes.

The most prominent mechanism for a false-positive GERD finding was technical error arising from factors that directly compromise sensor integrity, including 6 cases of dislodgement of the Bravo capsule into the stomach and upward transnasal catheter withdrawal each (Table 1). For instance, one such patient underwent a Bravo study for general symptoms of dyspepsia. On day 3 of the study, the capsule demonstrated a sudden, sustained decrease in esophageal pH to below 2 without expected physiologic variability or concomitant reflux symptoms. Though the patient had originally exhibited unremarkable pH readings, this severe drop below the standard pH threshold of 4 elevated the composite AET to a pathologic degree. Follow-up investigation by the clinician at the conclusion of the study revealed that the capsule was dislodged into the stomach and was instead recording the gastric environment, thus artificially inflating acid exposure and producing a false-positive GERD interpretation. Similarly, in a pH-impedance study, the esophageal channel abruptly displayed continuous deep-acid pH while the gastric channel became erratic. This phenomenon contributed to an elevated AET suggestive of severe acid reflux. However, this abnormal pattern initiated further follow-up, which revealed that the patient had intentionally pulled his catheter out and discarded it, thus inverting the sensor and producing a markedly prolonged esophageal AET before the sensor disconnected. Initial waveform evaluation, as opposed to sole reliance on AET, allowed clinicians to identify these abnormal shifts in esophageal pH, ultimately prompting a robust follow-up investigation to identify the mechanisms underlying these false-positive findings.

Esophageal stasis represents another source of bias that contributed to three false-positive diagnoses secondary to conditions such as achalasia and systemic sclerosis (SSc). Two patients with a past medical history of SSc had undergone a catheter-based pH study for chronic symptoms of dysphagia. Study findings revealed a pathologically elevated AET with prolonged distal esophageal acidification that exceeded even physiologic gastric acidity. Further diary review and waveform analysis demonstrated nocturnal reductions in intraluminal pH in the absence of corresponding gastric acidity or other symptomatic events. This pattern reflected esophageal dysmotility and subsequent intraesophageal reflux secondary to SSc, which resulted in bolus stasis and fermentation of retained carbohydrates within the distal esophagus. Though the abnormal AET appropriately aligned with decreased esophageal pH, the findings were not derived from gastric refluxate characteristic of GERD, thus potentially leading to a false-positive interpretation.

Lastly, five cases of esophageal behavioral disorders, including supragastric belching, gastric belching, and rumination syndrome, were identified as sources of an initial false-positive study. One patient underwent MII-pH for refractory dyspepsia and epigastric pain despite daily PPIs. Total AET was pathologically elevated with predominantly upright acidification and normal esophageal manometry findings. Waveform analysis revealed a recurring pattern of increased impedance followed by a reflux event that corresponded with a subsequent drop in impedance, suggestive of air influx pulling refluxate proximally. Notably, the rapid rise in impedance was most prominent in the proximal channels travelling distally toward the LES. The elevated AET was ultimately attributed to supragastric belching, in which the rapid swallowing and expulsion of air distend the LES and draw in gastric contents, thus generating symptoms of acid reflux. A similar reflux pattern was observed in three cases of gastric belching, which can be differentiated from SGB by its rise in impedance originating in the distal channels at the LES and moving proximally towards the oropharynx. The last patient was referred for a Bravo study due to chronic postprandial regurgitation and dyspepsia. pH findings again demonstrated elevated AET with recurrent esophageal acidification in concordance with symptomatic events. However, upon repeat testing with pH-impedance manometry, clinicians noted an intermittent increase in intra-abdominal pressure that directly preceded each reflux episode, which is highly characteristic of rumination syndrome. Thus, it was determined that the retrograde flow of acidic gastric contents was secondary to voluntary abdominal contraction and subsequent upward propulsion of refluxate, rather than true GERD. The transient esophageal acidification secondary to both esophageal dysmotility and esophageal behavioral disorders highlights the significance of not only discriminative modalities like impedance studies and manometry but also integration of clinical context into the diagnostic workflow.

## 4. Discussion

Esophageal pH monitoring is a cornerstone modality in GERD evaluation, but isolated, single-value interpretation of composite metrics like AET may misclassify patients when the diagnostic framework fails to account for potential confounders. This study highlights technical error, behavioral disorders, and physiologic comorbidities as impactful drivers of false-positive pH findings. While not directly observed in this study, improper PPI compliance, anatomic abnormalities, and patient-specific diet and activity may also contribute to GERD misclassification (Table 2). A structured, stepwise algorithm that incorporates the broader clinical context can contribute to minimizing these pitfalls.

### 4.1. Probe Positioning and Technical Artifact

Probe placement is highly dependent on clinician expertise, as well as both patient compliance and habits, thus rendering malposition as a significant source of error in pH studies. Dislodgement or migration of the pH sensor can be difficult to anticipate, as error may still arise despite appropriate insertion protocols and patient counseling. Patient-specific factors such as vigorous swallowing, coughing, retching, and inadvertent catheter manipulation can distally shift the probe and reflect gastric acid exposure, leading to a false-positive interpretation [23]. An abrupt, sustained drop in pH is characteristic of probe malpositioning rather than GERD. Unlike pathologic reflux episodes, which are transient and often accompanied by swallow-related clearance waves or impedance changes, dislodgement-related acidification tends to be prolonged and independent of physiologic esophageal recovery patterns. As such, review of the entire pH tracing is essential before integration of composite scores, ultimately allowing clinicians to better discriminate true reflux from technical artifact.

Orange juice swallows at the beginning of each study allow clinicians to characterize esophageal clearance, which can vary dramatically among patients [24]. Impedance recordings are typically derived from four segments within the distal esophagus and two segments from the proximal esophagus. Visualization of sequential bolus propagation across impedance channels then assists in localizing the LES during positioning of the pH sensor in MII-pH studies [25]. Probe insertion at the level of or distal to the gastroesophageal junction can result in inflated recordings of intragastric acidity, potentially contributing to a false-positive classification of pathologic reflux [25,26].

### 4.2. Physiologic Confounders

Esophageal motility constitutes another key parameter in pH monitoring; clearance of esophageal contents is diminished in conditions like achalasia and systemic sclerosis (SSc), in which prolonged contact of acidic material can spuriously inflate AET independent of reflux events. In patients with a known history or high clinical suspicion for dysmotility, esophageal manometry and HRIM are critical modalities for characterizing esophageal motor function and intraluminal conditions [27]. Motility disorders must be addressed before pH testing in order to identify confounding acid exposure secondary to abnormal esophageal clearance. Hypokinetic esophageal peristalsis in scleroderma can lead to dilation of the distal esophagus and a persistent food bolus presence that must be distinguished from GERD through pH impedance. Advanced SSc may present with low baseline impedance, thus limiting the utility of impedance testing; esophageal manometry should be performed in such patients to evaluate resting electrical and contractile activity [27]. Esophageal stasis and the subsequent fermentation of retained food residue result in a characteristic prolonged acidification within the distal esophagus, which may produce a false-positive reading for GERD if the underlying motility dysfunction is not recognized. On the other hand, the abnormally powerful peristaltic contractions of hypercontractile esophagus (HE) may potentially obscure pathologic reflux by rapidly clearing refluxate from the distal esophagus, producing a false-negative finding [28]. These implications further emphasize the value of HRIM for not only assessing esophageal motility disorders and contextualizing abnormal AET but also identifying the LES for accurate probe positioning.

Postprandial HRIM (PP-HRIM) serves as a valuable adjunct for all patients presenting with belching or regurgitation. PP-HRIM is both highly specific and sensitive for supragastric belching (SGB), which is confirmed via an anterograde elevation in impedance that precedes the reflux event as negative intraluminal pressure draws air into the esophagus [29]. While severe SGB is associated with concurrent GERD, these are distinct conditions that must be diagnosed independently [30,31]. SGB demonstrates a distinct impedance pattern that allows for differentiation from true GERD or similar conditions like gastric belching. The air sucking in SGB correlates with a rapid rise in impedance in the proximal channels moving aborally, while an impedance pattern that begins in the LES and travels proximally is more characteristic of gastric belching [32]. Additionally, perfect or near-perfect temporal correlation between regurgitation symptoms and reflux events strongly suggests rumination, as opposed to GERD [33]. Rumination syndrome is notably difficult to recognize, as diagnosis often requires postprandial waveform analysis, which is often excluded in pH studies. Detailed history collection may better enable diagnosis of rumination; for instance, clinical suspicion should be high if reflux events occur exclusively during the day. Belching and rumination can produce acid exposure profiles and characteristic heartburn that may otherwise be attributed to GERD, further emphasizing the importance of expanded diagnostic assessment beyond composite metrics to minimize false-positive outcomes [31]. Once identified, both disorders are often managed with behavioral modification, such as cognitive behavioral therapy, as opposed to anti-reflux intervention [30].

### 4.3. Pre-Test Evaluation and Anatomical Considerations

Anatomic abnormalities, specifically hiatal hernias, and inherent human variability in PPI compliance, diet, and activity are additional factors that may contribute to an overestimation of esophageal acid exposure [34,35,36,37]. While these confounders were not specifically observed in this present study, it is nonetheless important to employ a sound diagnostic framework that accounts for their potential to influence pH findings. Prior to each study, clinicians should confirm indication, as determining appropriate PPI status and dosing is a critical step in ensuring that study findings are interpreted within the correct context. A baseline gastric pH should also be measured during probe placement to confirm appropriate PPI adherence [38]. Patient diaries should also be reviewed to correlate findings with meal timing and content, as well as upright and recumbent periods [39].

Patients may be assessed for anatomic confounders through esophagogastroduodenoscopy (EGD) and review of prior medical history. Hiatal hernias are one such variable that can distort pH findings via “pocket effects,” which describe a reservoir of retained gastric contents that may re-enter the esophagus following the primary clearance wave, thus artificially elevating acid exposure [40,41,42]. Furthermore, surgical interventions such as bariatric procedures and fundoplication can significantly alter esophageal anatomy and complicate standard probe placement at the gastroesophageal junction [26,43].

## 5. Conclusions

False-positive esophageal pH studies arise from diverse mechanisms including catheter displacement, anatomical abnormalities, esophageal disorders, and physiologic or posture-dependent acidification. AET is often employed clinically in order to quantify GERD burden in a standardized manner, but composite scores cannot independently distinguish artifact from true reflux. Our findings reveal several indicators that the recorded AET may not reflect true reflux. These include markedly elevated esophageal AET, when esophageal acid exposure is longer than gastric exposure time, and exclusively upright reflux when accompanied by symptoms suggestive of belching or rumination. A structured diagnostic framework can contribute to reducing GERD misclassification and ensure appropriate management, as confirmation of data integrity and exclusion of confounders must precede interpretation of these metrics.

Prior to study onset, clinicians should not only evaluate esophageal anatomy and motility but also consider functional disorders that may alter reflux readings. Initial orange juice swallows and impedance recordings can facilitate accurate localization of the LES during probe placement, while a baseline gastric pH measurement may assist with verification of appropriate PPI adherence. Whole-study analysis of the pH tracing is essential for identification of abrupt pH shifts or prolonged acidification patterns suggestive of probe mispositioning, which may occur due to patient maneuvering or improper initial placement. Similarly, comprehensive review of the patient’s diary can offer valuable context for abnormal pH readings through discrimination of meal- or position-related acid exposure unrelated to true pathologic reflux. In the event of borderline or discordant findings, symptom correlation can complement traditional pH-derived metrics and provide additional insight into a patient’s reflux burden.

Prospective studies are necessary to fully determine the extent to which this proposed framework enhances diagnostic accuracy and improves long-term patient outcomes. These studies should assess the real-world feasibility of implementing such extensive adjunctive testing, as well as identify the measures that maximize clinical efficiency. Furthermore, additional investigation should also validate these workflows in special demographics, such as post-surgical patients with altered anatomy. More rigorous studies may further standardize patient-dependent variables, such as diet and body position, to better characterize their impact on pH findings and potentially inform patient instructions. This research may ultimately establish testing guidelines for specific populations in order to mitigate confounders associated with various comorbidities, symptom profiles, or patient behaviors.

## Figures and Tables

**Table 1:** Prominent false-positive pH patterns, including mechanism, waveform appearance, and corrective measures. HRM—high-resolution manometry.

Mechanism	Tracing	Pattern	Solution
Dislodged capsule	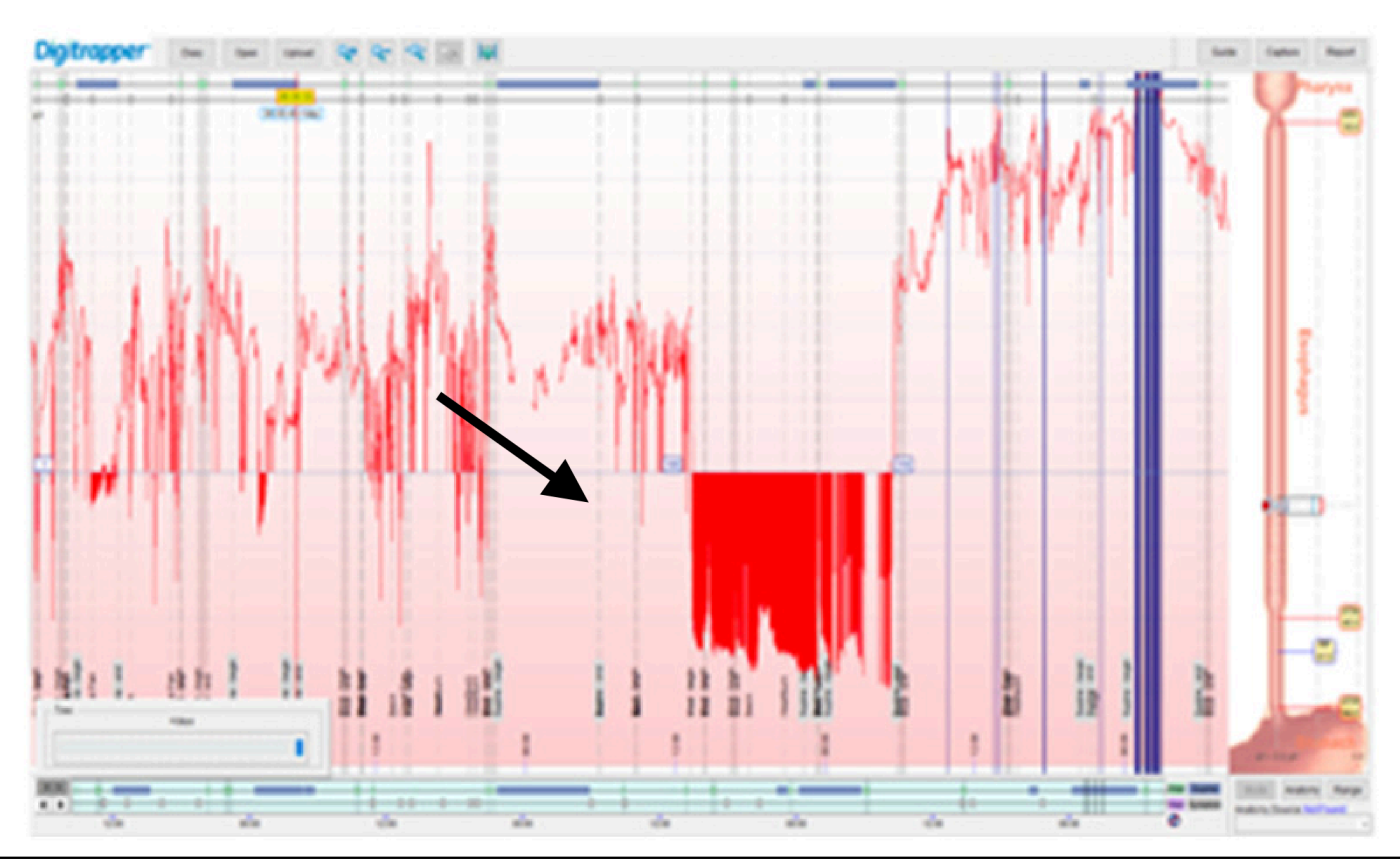	Sudden, sustained drop in esophageal pH to <2 with loss of physiologic variability	Confirm malposition; exclude study and repeat
Catheter withdrawal or inversion	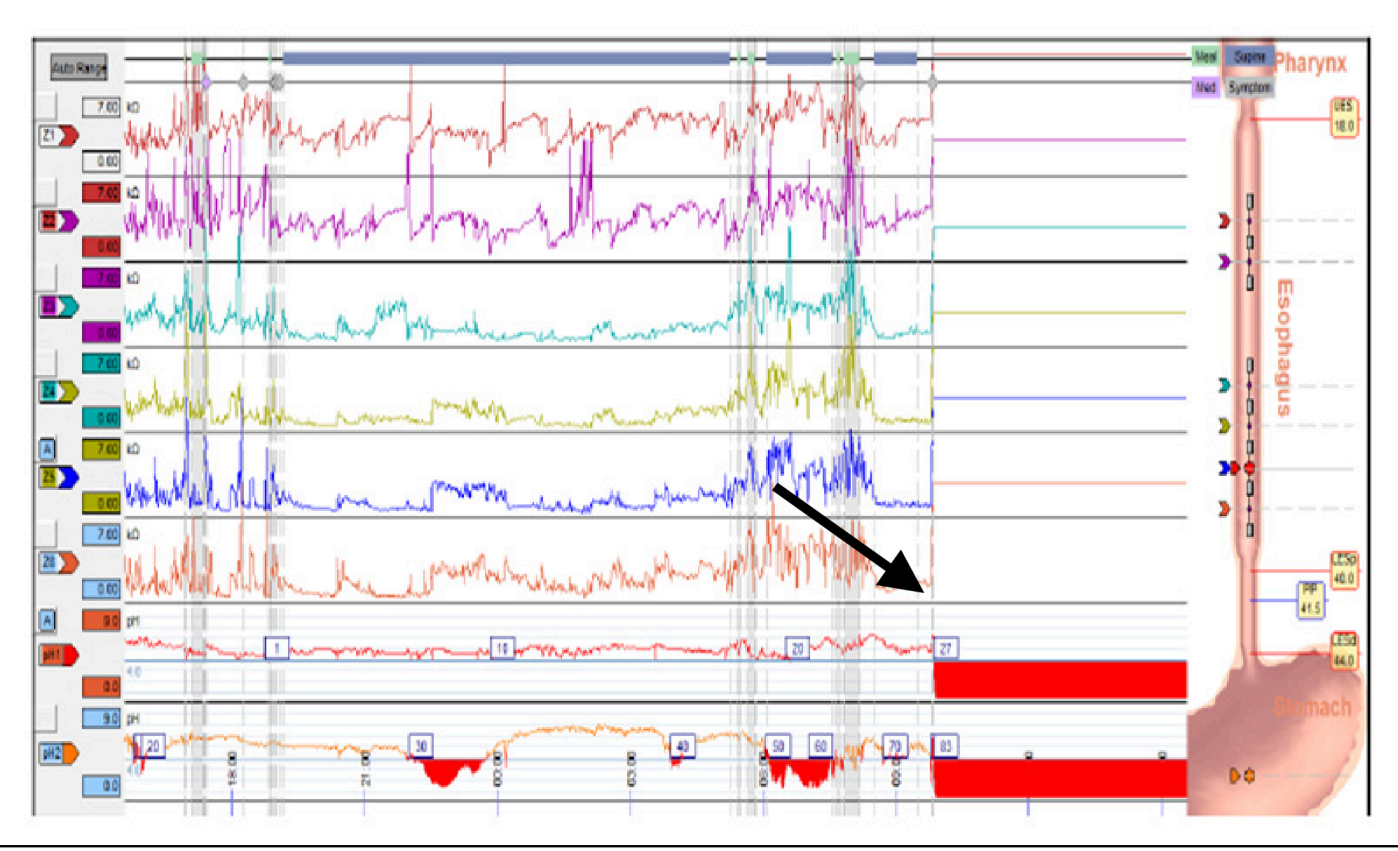	Deep acid pH on esophageal sensor with erratic gastric channel	Identify mechanical artifact; exclude study and repeat
Esophageal stasis	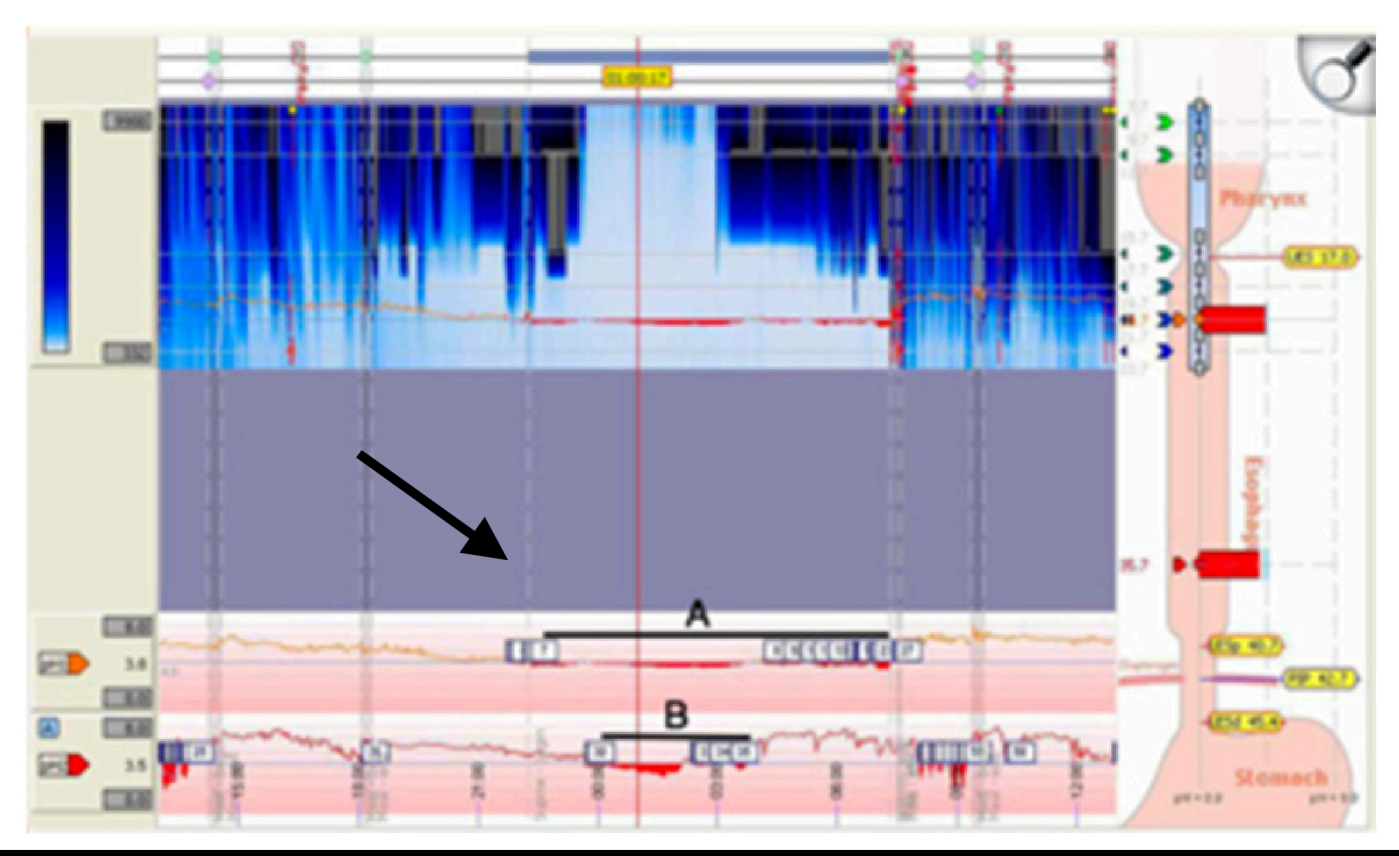	Esophageal pH lower than gastric pH; nocturnal worsening without reflux events	Evaluate for underlying motility disorder via HRIM; treat stasis/fermentation
Absent esophageal peristalsis	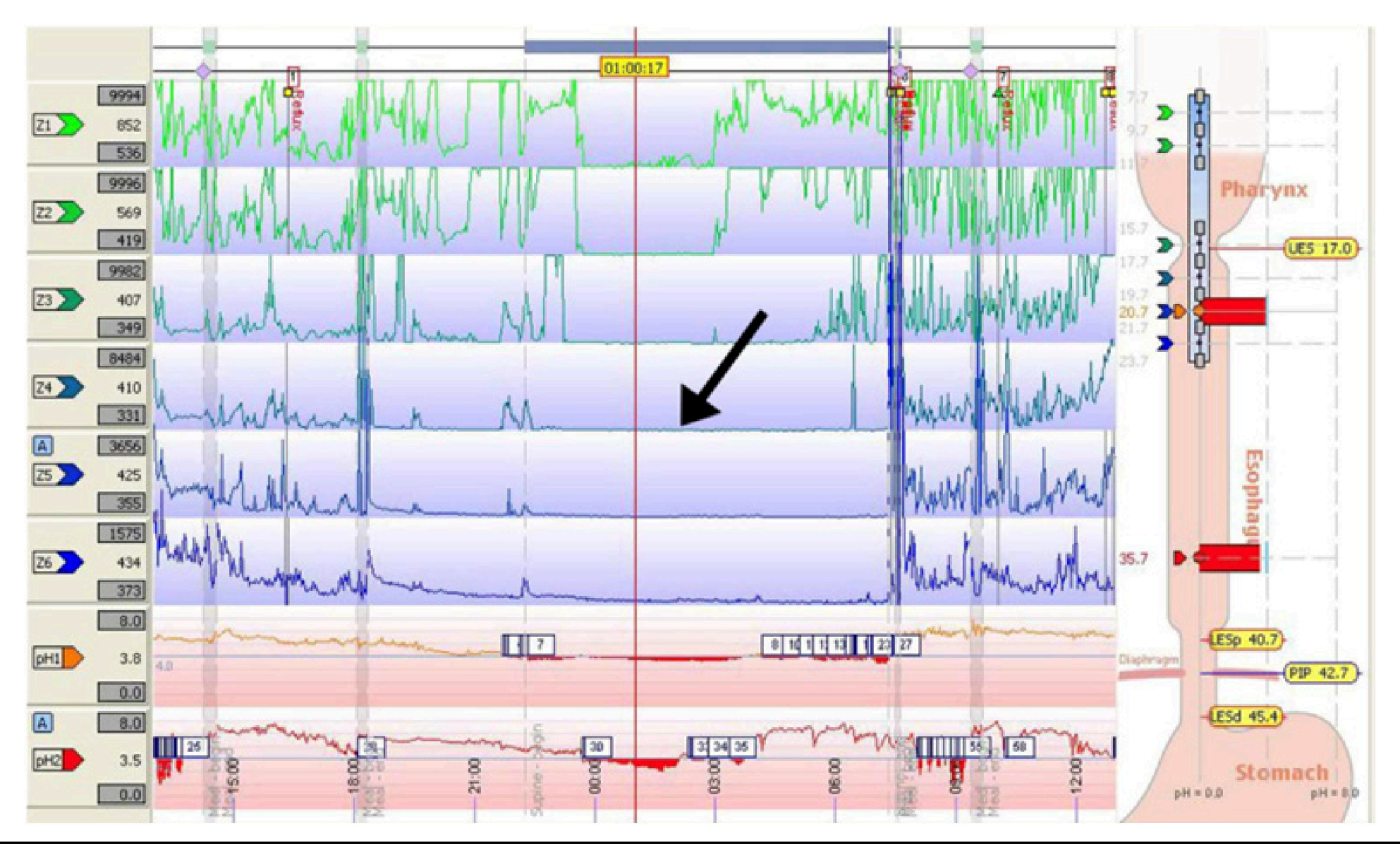	Absent oscillatory pattern of esophageal contractile waves [22]	Evaluate for underlying motility disorder via HRM
Air swallowing	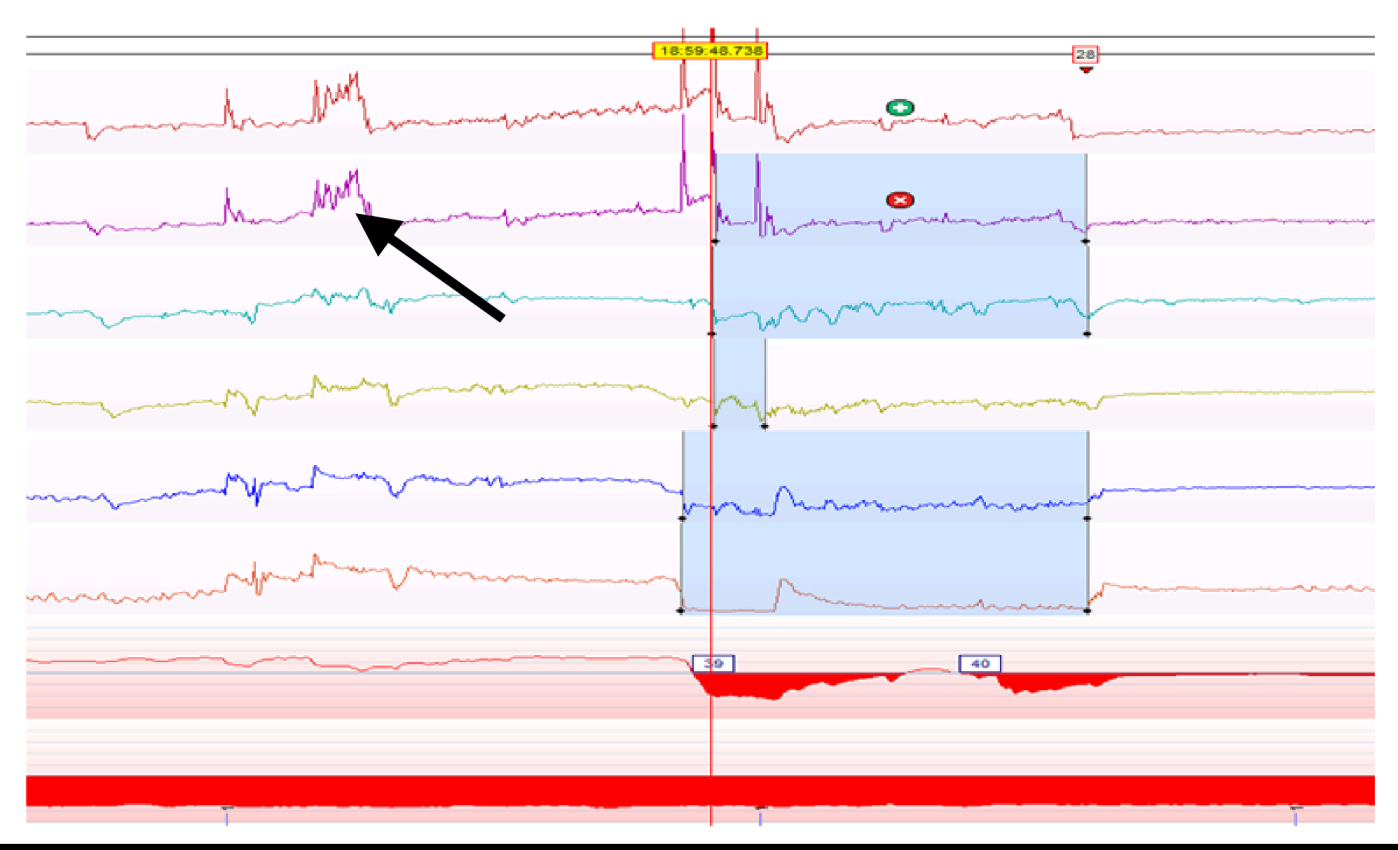	Increased impedance immediately preceding drop in pH	Re-analyze waveform to exclude any reflux signal that follows an air swallowing event

**Table 2:** Non-mechanical false-positive patterns in ambulatory pH or pH-impedance monitoring. This table offers a broad conceptual framework of mechanisms that may contribute to a false-positive pH finding. Of note, this table does not solely represent a strict summary of the cases observed in this study. PPI—proton pump inhibitor, EGD—esophagogastroduodenoscopy, HRM—high-resolution manometry, SLP—speech-language pathologist.

False Positive Mechanism	Study Findings	Confirmation	Solution
Dislodged capsule/ misplaced catheter	Gastric pH inconsistent with expected PPI status	Confirm distal probe depth, reassess placement	Repeat study if placement is uncertain; utilize adjunctive measures for precise position
Incorrect PPI status/compliance	Abnormal AET without symptom or diary correlation	Verify PPI adherence and expected gastric pH	Reinforce correct PPI indications, reinterpret with proper adherence
Anatomic abnormalities (hiatal hernia, altered foregut anatomy)	Stable low esophageal pH unrelated to reflux events or without expected variability	EGD, HRM, imaging	Interpret cautiously, evaluate anatomy
Behavioral disorders	Rapid oscillations, non-reflux impedance patterns	Impedance waveform review	Refer for behavioral therapy or SLP
Diet-related acidic artifact	Post-meal physiologic acidification	Diary correlation	Reinterpret as physiologic, not indicative of GERD
Unlogged posture changes	Recorded increase in acid exposure	Comparison of posture log with expected tracings	Adjust analysis for exclusion, reinterpret as physiologic

## Data Availability

The original contributions presented in this study are included in the article. Further inquiries can be directed to the corresponding author.

## Abbreviations

The following abbreviations are used in this manuscript:

AET	acid exposure time
EGD	esophagogastroduodenoscopy
GERD	gastroesophageal reflux disease
HE	hypercontractile esophagus
HRIM	high-resolution impedance manometry
LES	lower esophageal sphincter
MII-pH	multichannel intraluminal impedance-pH
PPI	proton pump inhibitor
RSI	reflux symptom index
SAP	symptom-associated probability
SGB	supragastric belching
SSc	systemic sclerosis
